# Translation and cross-cultural adaptation of *Adolescent Asthma Self-Efficacy Questionnaire* to Brazilian Portuguese and analysis of its measurement properties

**DOI:** 10.1016/j.clinsp.2025.100752

**Published:** 2025-08-26

**Authors:** Nathalia Ribeiro Berdu, Fernanda Lehrbaum, Adriano Luppo, Dirceu Solé, Gustavo Falbo Wandalsen, Karina Couto Furlanetto, Simone Dal Corso

**Affiliations:** a Graduate Program in Rehabilitation Sciences, Universidade Nove de Julho, São Paulo, SP, Brazil; b Graduate Program in Rehabilitation Sciences, Universidade Pitágora ‒ Unopar, Londrina, PR, Brazil; c Laboratory of Research in Respiratory Physiotherapy (LFIP), Department of Physiotherapy, Universidade Estadual de Londrina, Londrina, PR, Brazil; d Division of Allergy and Clinical Immunology, Department of Pediatrics, Universidade Federal de São Paulo (UNIFESP), São Paulo, SP, Brazil; e Respiratory Research@Alfred, Central Clinical School, Monash University, Level 5, Melbourne, Australia

**Keywords:** Self-efficacy, Asthma, Questionnaires, Quality of life

## Abstract

**Introduction:**

Some scales evaluate the self-efficacy of asthmatic adolescents, but do not consider issues related to age and the correct use of medication.

**Goal:**

To translate to cross-culturally adapt to Portuguese and to test the psychometric properties of the Adolescent Asthma Self-Efficacy Questionnaire (AASEQ).

**Methods:**

The questionnaire was translated and adapted to Portuguese. The final version was applied in two moments with an interval between seven and fourteen days. Asthma-related Quality of Life Questionnaires (PedsQL and PAQLQA) were also applied. Reliability, agreement, construct validity, ceiling and floor effect, and confirmatory factor analysis were evaluated.

**Results:**

A total of 135 adolescents (median age 14 (interquartile range 12‒16) were evaluated, and the severity of asthma was mild in 63 (47 %), with controlled symptoms in 90 (67 %). The internal consistency of the AASEQ was adequate (Cronbach's alpha 0.70). The standard error of measurement was very good (SEM = 5.82 points and MDC90 % = 6.68), with moderate reliability (ICC_3,1_: 0.68 [95 % CI: 0.55–0.77], *p* < 0.0001. The validity of the AASEQ construct was poor with PedsQL (*r* = 0.28 and *p* < 0.05) and PAQLQA (*r* = 0.27 and *p* > 0.05). The floor and ceiling effects were adequate.

**Conclusion:**

AASEQ is a reliable tool to assess self-efficacy in Brazilian adolescents with asthma.

## Highlights

• AASEQ is a self-efficacy tool with rigorous validation procedures.
• AASEQ was translated and cross-culturally adapted to Brazilian Portuguese.
• AASEQ shows good internal consistency and substantial test-retest reliability.
• AASEQ fills a gap in assessing self-efficacy in Brazilian asthmatic adolescents.

## Introduction

Asthma is a chronic inflammatory airway disease that commonly affects adolescents.^1^ In Brazil, there are approximately five deaths per day and more than 120,000 hospitalizations per year due to asthma. However, in the last six years, there has been a 10 % reduction in deaths and 36 % fewer hospitalizations due to asthma.^2^ As a consequence, asthmatic adolescents have a higher rate of school truancy.^3^ Despite the availability of modern and effective therapies for asthma, many adolescents have suboptimal control of the disease,^4^ with consequent impairment of quality of life.^5^ A study by Birkhead et al.^6^ showed that a common point in the death of 5 adolescents with asthma was poor adherence to treatment. The Brazilian average prevalence of active asthma is 22.7 % for adolescents, with a worldwide average ranging from 1.8 % to 36.7 %.^6^

In health, self-efficacy has been studied to understand how individuals promote their health or face a disease process. Studies show that the level of self-efficacy determines the outcome of activities that are aimed at preserving or regaining health.^7^^,^^8^ There is evidence that low levels of self-efficacy are related to negative perceptions of patients concerning their ability to adopt self-care or well-being behaviors, and may have as an outcome the belief that taking care of their health is outside their individual capacities.^9^

Some scales evaluate asthmatic adolescents' self-efficacy, but all have limitations. In a study conducted by Van Es et al.,^10^ questionnaires based on the concepts of the Attitude-Social Influence-Self-Efficacy model (ASE-model),^11^^,^^12^ in which the concepts of behavior, social influences, and self-efficacy in adolescents with asthma were evaluated. They were divided into control groups (without intervention) and intervention groups, and the intervention was based on an educational program. There was only a statistical difference in the evaluation after 24 months from the initial evaluation, in which the intervention group showed greater self-reported adherence compared to the control group. The authors believe that one possible reason for the result was that the variables evaluated in the scale used were not the most relevant for that age, and the most pertinent variables have not yet been found. The Self-Efficacy Scale for children and adolescents with asthma, was the first developed to assess self-efficacy in asthmatic children. Despite evaluating three important concepts, such as medical treatment, environment, and problem-solving, it does not evaluate beliefs about specific self-management behaviors that directly impact the prevention and control of symptoms, such as, for example, the correct use of an inhaler.^13^ Both instruments cited above has been translated into Brazilian Portuguese, nor have cross-cultural adaptation and evaluation of their clinometric properties been made.

The Adolescent Asthma Self-Efficacy Questionnaire (AASEQ) is the first self-efficacy questionnaire developed especially for adolescents aged 12 to 18 that uses robust and recommended specific methodology. For its development, an extensive literature review was carried out, and specific groups of adolescents, parents, and professionals in the area were interviewed, in addition to established test-retest reliability and construct validity.^14^

Thus, the present study aimed to translate, cross-culturally adapt, and test the Adolescent Asthma Self-Efficacy Questionnaire (AASEQ) measurement properties in adolescents between 12 and 18 with an asthma diagnosis. The authors hypothesize that the AASEQ will demonstrate adequate measurement properties for use with Brazilian adolescents.

## Method

Data collection was performed at the Allergy, Clinical Immunology and Rheumatology outpatient clinic of the Department of Pediatrics of the Federal University of São Paulo, as well as at the Hospital das Clínicas in Londrina, and was conducted between March 2021 and November 2022. This study followed the STROBE Statement checklist. Eligible participants were approached by the researchers, and verbal and written study information was provided by them. Those responsible for the adolescents signed the Informed Consent Form, while the adolescents also signed the Assent Form. The Ethics and Research Committee of the Nove de Julho University (n° 33873520.9.0000.5511), Federal University of São Paulo (n° 33873520.9.3001.5505), and University North of Paraná (n° 33873520.9.2002.0108) approved the study. All the procedures were conducted in line with ethical principles outlined in the Declaration of Helsinki and local research ethics guidelines.

The participants were adolescents between 12 and 18 years old, who were Brazilian natives, with a parent or legal guardian present at the appointment, who had a clinical asthma diagnosis without exacerbations in the four weeks prior to entry into the study.^15^ In addition, they should be on regular use of prophylactic medication. Patients with rhinosinusitis, eczema, or food allergy were not excluded due to the high association between such morbidities and asthma.^16^^,^^17^ Adolescents with cognitive impairments and those with other pulmonary diseases such as cystic fibrosis, tuberculosis, and sequelae of COVID-19 were excluded from the study.

The study was conducted in two phases. In Phase I, the original version of the AASEQ was translated and cross-culturally adapted into Brazilian Portuguese following previously established guidelines.^18^ The initial translation was carried out by two independent bilingual translators residing in Brazil, whose native language is Brazilian Portuguese and English as a second language, one of whom had specific knowledge in the field and the other was lay. The two translated versions were compared and combined by the translators to produce the first Brazilian version of both instruments. This version was then retranslated into English by two independent bilingual translators without prior knowledge and no access to the original version, as well as lacking specific knowledge in the field. Following this phase, a panel of experts composed of two pulmonologists and two physiotherapists formulated a final version through comparison among the original version, translations, and back-translations. The final version was administered to a sample of 35 participants. The participants in this pilot study were invited to complete a feedback form aimed at identifying potential issues with questionnaire items. They were asked to specify any item they found challenging to answer and to indicate whether they experienced difficulties such as lack of clarity, confusing wording, unfamiliar terminology, or discomfort. Additionally, they were prompted to provide suggestions for rephrasing the item by responding to questions such as: “How would you paraphrase this item?” and “How would you formulate this question?”. Following this analysis, the final version was forwarded and approved by the authors of the original questionnaire.

In Phase II, the measurement properties were tested. After meeting the inclusion and exclusion criteria, participants responded to the AASEQ-1 (Test), followed by the questionnaires Pediatric Quality of Life Inventory (Peds-QL) and Pediatric Asthma Quality of Life Questionnaire (PAQLQ-A). After 7 to 14 days, the subjects answered the AASEQ-2 (retest) again, administered in the form of an interview, through a phone call. This modality of application was chosen and accepted as a methodological bias due to the participants not being able to attend the outpatient clinic again, given the global COVID-19 pandemic. To minimize the impact on the study, 42 adolescents answered the questionnaire in reverse order; that is, the phone application was performed at the time of the test, and in person at the time of the retest.

The term sex was used to refer to a set of biological attributes that are associated with physical and physiological features, categorizing into male or female.

Anthropometric data were measured and analyzed through the *z*-score, according to World Health Organization recommendations.^19^ Body weight was measured on a Welmy® anthropometric mechanical scale. In addition, asthma severity and symptom control of asthmatic adolescents were collected.^15^

Spirometry was used to classify the pulmonary function of the research participants. Their last spirometry performed was recorded. Initially, in the absence of such a test, or if the last one was performed before six months, spirometry would be scheduled to be done again in the outpatient clinic, by a trained and qualified professional. However, because of the limitations imposed on performing this complementary test by the COVID-19 pandemic, the last spirometry taken by the participants was considered. Spirometry was performed according to the guidelines for Pulmonary Function Tests provided by the Brazilian Society of Pulmonology, as well as respecting their contraindications.^20^ The parameters obtained by spirometry were Forced Vital Capacity (FVC), FEV_1_, FEV_1_/FVC ratio, and forced expiratory flow between 25 % and 75 % of the FVC curve (FEF_25‒75 %_).^15^^,^^20^

### Adolescent Asthma Self-Efficacy Questionnaire

In order to develop it, an extensive literature review was carried out by Holley et al.,^14^ specific groups of adolescents, parents, and professionals in the area were interviewed, and also to establish test-retest reliability and construct validity. The questionnaire presents 27 items, subdivided into four domains: medication, symptom management, beliefs about asthma, and friends, family, and school. On each item questioned, the adolescent must answer, on a scale of 0 to 100, how confident he/her feel, with 0 meaning “cannot do it” and 100 “certainly can do it”. All the answers are added, and the total value is divided by 27, obtaining a result that varies between 0–100, and the higher the value obtained, the better the self-efficacy in asthma management. Authorization was given by the authors of the original instrument, and the original questionnaire can be found in the Supplementary Material.

### Statistical analysis

The sample size was determined following the COSMIN guidelines, which recommend an adequate sample size of at least five times the number of items in the questionnaire. Since the AASEQ comprises 27 items, the authors utilized a sample of 135 participants. The normality of the data was investigated using the Shapiro-Wilk test. The Wilcoxon test was used to compare AASEQ-1 and AASEQ-2 scores. The significance level was set at 5 % (Two-Tailed) for all analyses. Cronbach's Alpha was used for internal consistency analysis. The Intraclass Correlation Coefficients (ICC_3,1_) and 95 % CI were calculated in the test-retest reproducibility analysis. The agreement was analyzed using the Standard Error of Measurement (SEM) (SEM = SD√1-ICC) and interpreted as very good (≤ 5 %), good (5 % to 10 %), questionable (11 % to 20 %), and poor (> 20 %). The minimum difference detected with 90 % confidence was calculated using the EPM using the following formula: SDC=1.96×√2×EPM.^21^^,^^22^ Agreement was also analyzed using the Bland-Altman plot. Spearman's correlation coefficient confirmed the concurrent validity between AASEQ, Peds-QL, and PAQLQ-A scores. The ceiling and floor effects were measured by calculating the percentage of patients indicating the maximum (ceiling) or minimum (floor) possible scores considered present if 15 % of patients or more achieve the maximum or minimum score of the questionnaires, respectively.^21^ SPSS Statistics (version 21; IBM) was used for such tests. Confirmatory factor analysis was performed using JASP (version 16.4; University of Amsterdam). The sample size was adequate, following previously established guidelines for statistical analysis.^23^, ^24^, ^25^

## Results

During the phase of cross-cultural adaptation, there were no sentences of the AASEQ that required changes of descriptive terms since the terms and situations described have their respective similarities in Portuguese. Following the administration of the questionnaire in the pilot study, no adolescents reported experiencing difficulties, confusion, complex wording, or discomfort, nor were any suggestions made to modify any of the questionnaire items. Therefore, no adjustments to the instrument were deemed necessary. The final version of the questionnaire is available at the Supplementary Material.

A total of 160 adolescents were included in the study. No adolescents had asthma exacerbation during the test and retest period, there was no need for exclusion from the study; 17 adolescents did not respond to the retest because they either didn't answer the phone call that would apply the questionnaire or their phone number was unavailable; two adolescents had another associated lung disease, and six adolescents had chronic disease with cognitive impairment. Therefore, the data of 135 adolescents are presented in Table 1. Of these, 73 were evaluated in São Paulo/SP and 62 in Londrina/PR.

**Table 1:** Characteristics of the participating adolescents (*n* = 135).

Characteristics	Values
Place of assessment, n ( %)	
São Paulo	73 (54)
Londrina	62 (46)
Age^b^	14 (12–16)
Age of enrollment in the outpatient clinic (in years)^a^	8 ± 4
Age of onset of asthma (in years)^b^	3 (1 6)
Age of diagnosis of asthma (in years)^b^	4 (2–7)
Sex, n ( %)	
Male	76 (56.3)
Female	59 (43.7)
Weight (kg)^a^	55.37 ± 15.34
Height (m)^b^	1.58 (1.51 – 1.67)
Height-for-age, n ( %)	
Height appropriate for age	126 (93.3)
Very low height-for-age	6 (4.4)
Low height-for-age	3 (2.2)
BMI kg/m^2^^a^	21.3 (18.8 – 25.7)
BMI-for-age, n ( %)	
Eutrophy	120 (88.9)
Overweight	11 (8.1)
Obesity	2 (1.5)
Severe obesity	2 (1.5)
Race, n ( %)	
White	90 (66.7)
Brown	28(20.7)
Black	17 (12.6)
Adolescent education, n ( %)	
8th Grade elementary school	34 (25.5)
6th Grade elementary school	19 (14.1)
7th Grade elementary school	17 (12.6)
1st Year high school	15 (11.1)
9th Grade elementary school	14 (10.4)
3st Year high school	13 (9.6)
5th Grade elementary school	12 (8.9)
2st Year high school	11 (8.1)
Resides in urban area, n ( %)	128 (94.8)
Asthma severity, n ( %)	
Mild	63 (46.6)
Moderate	51 (37.8)
Severe	21 (15.6)
Symptom control, n ( %)	
Well-controlled	90 (66.7)
Partly controlled	33 (24.4)
Uncontrolled	12 (8.9)
Self-reported forgetfulness of preventive medication, n ( %)	
Occasionally	52 (38.5)
Never	36 (26.7)
Once a week	24 (17.8)
Half the time	18 (13.3)
All the time	3 (2.2)
Most of the time	2 (1.5)
Asthma triggers, n ( %)	
Dust	109 (80.7)
Weather	103(76.3)
Colds	79 (58.5)
Emotions	68 (50.4)
Cigarette smoke	66 (48.9)
Animals	42 (31.1)
Soaps	40 (29.6)
Pollen	29 (21.5)
Vapors	16 (14.1)
Food	18 (13.3)
Associated allergies, n ( %)	
Rhinitis	105 (77.8)
Allergy to animals	40 (29.6)
Food allergy	32 (23.7)
Eczema	27 (20)
Number of exacerbations in the last 12-months^b^	0 (0 – 2)
Emergency room visits in the last 12-months^b^	0 (0 – 1)
Number of total hospital visits^b^	3 (0 ‒ 10)
Charlson index^b^	0 (0 ‒ 0)
Performed cardiorespiratory rehabilitation, n ( %)	7 (5.2)
Peds-QL^b^	79.35 (66.3 – 89.4)
PAQLQ-A^b^	5.69 (4.99 – 6.27)
FVC, L^a^ / %pred^a^^,^^c^	2.8 ± 0.8 / 98.5 ± 13.4
FVC post-BD, L^a^ / %pred^a^^,^^c^	3.0 ± 0.8 / 102.1 ± 14.5
FEV_1_, L^a^ / %pred^a^^,^^c^	2.3 ± 0,6 / 91.0 ± 17.5
FEV_1_ post-BD, L^a^ / %pred^a^^,^^c^	2.4 ± 0.7 / 97.3 ± 16.6
FEV_1_ / FVC, L^a^ / %pred^a^^,^^c^	80.5 ± 10.6 / 91.9 ± 12.6
FEV_1_ / FVC post-BD, L^a^ / %pred^a^^,^^c^	81.3 ± 14.1 / 95.0 ± 12.4
FEF_25–75 %_, L^a^ / %pred^a^^,^^c^	3.0 ± 1.6 / 76.3 ± 28.7
FEF_25–75 %_ post-BD, L^a^ / %pred^a^^,^^c^	3.3 ± 1.5 / 100.7 ± 123.0

a Data expressed as mean ± standard deviation.

b Data expressed as median (interquartile range).

c *n* = 69. n, Number of participants; %, Percentage; AASEQ, Adolescent Asthma Self-Efficacy Questionnaire; Peds-QL, Pediatric Quality of Life Inventory; PAQLQ-A, Pediatric Asthma Quality of Life Questionnaire; FVC, Forced Vital Capacity; FEV_1_, Forced Expiratory Volume in one second, FEV_1_/FVC ratio; FEF_25–75 %_, Forced Expiratory Flow between 25 % and 75 % of the FVC curve; BD, Bronchodilator; pred, Predicted.

Spirometry was collected from the participating adolescents, and the variables are shown in Table 1. Due to the COVID-19 pandemic and its restrictions, 66 adolescents did not perform the test.

The AASEQ scores at the two application moments are described in Table 2. Differences between the test and retest were found in the total score of the AASEQ, as well as in the medication, symptom control, and knowledge domains.

**Table 2:** Comparison of AASEQ scores during test and retest application.

Questionnaire	Test	Retest	p
AASEQ^a^	87.2(77.8 – 93.3)	89.6 (80.4 – 95.2)	<0.001
Subscales			
Medication^a^	92 (74 – 100)	94 (78 – 98)	0.04
Symptoms Control^a^	83.2 (65 – 91.3)	88.8 (75 – 93.8)	<0.001
Knowledge about asthma^a^	90 (80 – 96)	94 (86 – 100)	0.005
Friends, family and school^a^	94.4 (83.3 – 100)	95.6 (86.7 – 100)	0.27

a Data expressed as median (interquartile range); Wilcoxon test performed.

The AASEQ presented adequate internal consistency, with a Cronbach's Alpha of 0.7. The ICC_3,1_ was considered good and demonstrated substantial test-retest reliability (Table 3). The Bland-Altman plot arrangement showed limits of agreement of −22.76 to 17.7, with an average of differences of −2.53.

**Table 3:** Classification of AASEQ measurement properties in Brazilian Portuguese in patients with asthma (*n* = 135).

Properties	Values	Classification
**Internal consistency**		
Cronbach's Alpha	0.70	Substantial
**Measurement error**		
Standard error of measurement	5.82	Very good
Minimum difference detected	6.68	
**Reproducibility**		
ICC_3,1_ (95 % CI)	0.68 (0.55 – 0.77)^a^	Moderate
**Construct validity**		
PedsQL	(*r*) = 0.28^a^	Weak
PAQLQA	(*r*) = 0.27^a^	Weak
**Ceiling effect**	5.18 %	Adequate
**Floor effect**	absent	Adequate

ICC, Intraclass Correlation Coefficient; 95 % CI, 95 % Confidence Interval; R, Spearman correlation; AASEQ, Adolescent Asthma Self-Efficacy Questionnaire; Peds-QL, Pediatric Quality of Life Inventory; PAQLQ-A, Pediatric Asthma Quality of Life Questionnaire.

a *p* < 0.001.

A weak correlation was found between AASEQ and PedsQL scores, and in the correlation between AASEQ and PAQLQ-A scores (Table 3).

In the confirmatory factor analysis, the KMO coefficient was 0.780, and Bartlett's sphericity test was significant (*p* < 0.001).

Table 4 presents the internal consistency values of the AASEQ and its subscales. An acceptable Cronbach’s Alpha was found for all subscales, except for the “Friends, Family, and School” subscale. The intraclass correlation coefficient was moderate across all subscales.

**Table 4:** Internal consistency of the AASEQ and its subscales (*n* = 135).

Questionnaire	Cronbach's Alpha	ICC_3,1_ (95 % CI)
AASEQ	0.70	0.68 (0.55 – 0.77)
Subscales		
Medication	0.72	0.72 (0.61 – 0.80)
Symptoms Control	0.70	0.68 (0.54 – 0.78)
Knowledge about asthma	0.71	0.70 (0.58 – 0.78)
Friends, family and school	0.51	0.51 (0.31 – 0.65)

ICC, Intraclass Correlation Coefficient; 95 % CI, 95 % Confidence Interval, AASEQ, Adolescent Asthma Self-Efficacy Questionnaire; * *p* < 0.001.

## Discussion

AASEQ is a questionnaire recently developed to assess the self-efficacy of adolescents with asthma, but its Portuguese version was not available for use in Brazilian adolescents with asthma. The present study stands out for translating the AASEQ into Portuguese and testing its clinimetric properties.

Regarding the characteristics of this sample, the higher percentage of males corroborates the higher prevalence of asthma in male adolescents at this age, being up to two times higher than in female adolescents up to the age of 14 years.^15^ Among the associated allergies, the high incidence of rhinitis stands out, with 77.8 %. Previous studies have reported that rhinitis is associated with a high risk of developing asthma and more severe asthma, and this fact becomes more evident when there is also the presence of atopic dermatitis,^26^^,^^27^ which was present in 20 % of the adolescents in the present study.

The present study revealed differences in the internal consistency and reproducibility of the AASEQ compared to the original study. This finding may be because the test and retest application occurred in two ways, one in person and the other by phone, since the study was conducted during the COVID-19 pandemic, and the adolescent and guardian could not return to the outpatient clinic to perform the retest. In the present study, the authors had a sample of 135 adolescents who performed the test and a retest. In contrast, in the original study,^14^ only 34 % of the initial sample of 183 adolescents 63 completed the retest. Another issue to consider is the cultural and activity differences between countries. In the present study, all adolescents were recruited in a specific outpatient clinic of third-party hospitals, with a diagnosis of asthma established by a pulmonologist/allergist. In contrast, in the original study, some adolescents were recruited online, possibly creating selection bias.

Because there is no gold standard instrument in Brazilian literature used to assess the self-efficacy of asthmatic adolescents, the authors opted for using two instruments, the PedsQL and PAQLQ-A, which aim to assess the quality of life that can be impacted by self-efficacy. Both are translated and validated tools for use in Brazilian adolescents^28^^,^^29^ and are intended to assess adolescent quality of life, with PAQLQ-A being specific for asthmatic adolescents and PedsQL for use in adolescents with chronic pathologies. Several questionnaires evaluate self-efficacy in asthmatics, yet they pose drawbacks in their suitability in the validation process of the AASEQ. The scale “the Knowledge, attitude, and Self-efficacy Asthma Questionnaire (KASE-AQ)” is not specific for asthmatic adolescents and has no validation of its measurement properties in the Brazilian literature.^30^ The instrument “the Self-Efficacy Scale for children and adolescents with asthma”^13^ is aimed at asthmatic children and adolescents but does not incorporate crucial elements such as self-management of crises and appropriate use of medications, both linked to self-efficacy. Additionally, this scale has not yet been validated for use in Brazil. Another tool, “Self-efficacy and their child's level of asthma control”, seeks to assess self-efficacy in asthmatic children through parents and caregivers, not focusing directly on adolescents.^31^ Despite being weak, there was a correlation between the instruments, demonstrating that the AASEQ shows good construct validity. In the agreement analysis, an SEM of 5.82 was obtained, with an MDD of 6.68, values which are considered low in a questionnaire in which scores range from 0 to 100 points. Such an analysis was not performed in the questionnaire development study.

In a study by Sleath et al.,^32^ adolescents with higher reported self-efficacy were more likely to have a higher quality of life and controlled asthma, findings that are consistent with the results of the present study, in which high scores in self-efficacy and quality of life were observed, with a higher incidence of controlled asthma. Also, the positive correlation between quality of life and condition control is already documented,^33^ since fewer symptoms and exacerbations, less use of rescue medications, and fewer nocturnal awakenings are observed in a controlled condition.

The structural validity of the AASEQ was supported by confirmatory factor analysis, with a KMO coefficient of 0.780 and a significant Bartlett’s test (*p* < 0.001), indicating that the data were suitable for factor analysis. The overall internal consistency of the scale was acceptable, with a Cronbach’s alpha of 0.70, and test-retest reliability was demonstrated through a good ICC₃_,_₁ and a Bland-Altman plot showing limits of agreement between −22.76 and 17.7, with a small mean difference of −2.53. However, some limitations in structural validity emerged at the subscale level. Although most subscales demonstrated acceptable internal consistency, the “*Friends, Family, and School*” subscale showed a Cronbach’s alpha below the acceptable threshold, suggesting potential concerns regarding the coherence of items within this domain. Additionally, all subscales exhibited only moderate intraclass correlation coefficients, indicating that the temporal stability of the constructs measured by each subscale may be limited. Furthermore, weak correlations were observed between the AASEQ and external constructs such as the PedsQL and PAQLQ-A, which may suggest issues with convergent validity and raise questions about whether the subscales are capturing distinct, meaningful constructs aligned with related measures. In summary, while the AASEQ demonstrated acceptable overall psychometric properties, the findings point to limitations in the reliability and structural integrity of specific subscales, particularly “*Friends, Family, and School*”.

In terms of testing modalities, the questionnaire was administered with a 7- to 14-day interval to assess test-retest reliability. While this interval is considered acceptable for evaluating temporal stability, factors such as the testing environment, level of assistance provided, and participants’ familiarity with the questionnaire may have influenced the consistency of responses. Ensuring standardized administration conditions in future studies may help minimize these sources of variability and enhance the precision of reliability estimates.

The present sample consisted of adolescents with asthma recruited from two specialized outpatient clinics affiliated with universities. While the inclusion of participants from two distinct urban centers offers an improvement over single-site studies, the sampling frame remains restricted to institutional, urban contexts with access to highly specialized care. Adolescents receiving care in such settings are likely to benefit from enhanced clinical, educational, and multidisciplinary support, which may lead to higher reported levels of self-efficacy. Additionally, despite regional variation, the sample may exhibit relative socioeconomic and educational homogeneity due to the public nature of the services. These characteristics may limit the generalizability of the findings to broader adolescent populations, particularly those in rural or underserved areas with limited access to specialized asthma care. Consequently, caution is warranted when extrapolating these results. Future research should aim to recruit more diverse and representative samples, encompassing varied sociocultural contexts and levels of healthcare access, to strengthen the external validity of the instrument. The representativeness of the sample may influence the instrument’s performance across different testing modalities. Variations in participant profiles, such as clinical complexity, socioeconomic status, and educational background, combined with contextual factors inherent to diverse settings, can affect response patterns and engagement with the instrument. Adolescents assessed in specialized outpatient clinics may demonstrate higher comprehension and more consistent responses compared to those evaluated in primary care or community settings. It is essential to conduct further validation studies encompassing a wider range of populations and testing conditions to confirm the robustness and generalizability of the instrument’s psychometric properties.

A potential limitation of this study is that the relatively homogeneous sample may not fully capture the diversity of the general Brazilian asthma population. While some ethnic groups within the asthma population might have been underrepresented, the sample largely reflected observed sex differences. As this study was a cross-sectional survey, longitudinal analysis was not conducted, highlighting the need for a specific study to assess the instrument's stability over time. Patients were mainly recruited from specialized healthcare centers in Brazil. Consequently, the questionnaire responsiveness may vary for asthma patients in general primary healthcare settings. While the study demonstrates that the AASEQ is a reliable tool for assessing self-efficacy among Brazilian adolescents with asthma, several limitations must be acknowledged. First, the poor construct validity observed with both the PedsQL and PAQLQA suggests that the AASEQ may not strongly correlate with quality-of-life measures, which could be due to differences in the constructs measured by these tools. Self-efficacy focuses on the adolescents’ confidence in managing asthma, whereas quality-of-life measures encompass broader aspects of health and well-being. This limitation highlights the need for further research to clarify the relationship between self-efficacy and other relevant constructs. The authors did not evaluate whether parents or caregivers had asthma, a fact that could influence knowledge about the disease and perhaps the self-efficacy of asthmatic children. The ability of the scale to measure change over time in response to an intervention to improve self-efficacy in asthma management should be investigated. Due to the COVID-19 pandemic, it was not possible to apply the questionnaires in the same manner at two time points. In one moment, it was applied in person, and in the second, it was applied by a phone call. The authors alternated the application order in part of the sample to minimize methodological bias. In addition, not all adolescents underwent spirometry. Due to the contraindications imposed by the World Health Organization regarding the risk of virus spread, spirometry was performed only by adolescents with medical indications. Those with stable disease did not undergo the test. Another potential bias is related to the sample characteristics. Most participants had mild asthma (47 %) and controlled symptoms (67 %), which may limit the generalizability of the findings to adolescents with moderate or severe asthma or those with poorly controlled symptoms. This skew in the sample may have impacted the psychometric evaluations, particularly in capturing the full range of self-efficacy experiences in adolescents with diverse asthma severities. Additionally, participants with better asthma control may have inherently higher self-efficacy, introducing a bias that could overestimate the reliability or validity of the instrument.

AASEQ is a reliable and valid tool for adolescents with asthma. Self-efficacy is essential in managing long-term conditions, so AASEQ may be helpful in assessing the self-management of asthmatic adolescents. For health professionals working with asthmatic adolescents, the instrument can help evaluate interventions in the adolescent's routine.

## Funding

This work received funding from CAPES (Process 88887.613082/2021-00).

## Declaration of competing interest

The authors declare no conflicts of interest.

## Appendix Supplementary materials

Image, application 1
