# Supplementary material for: Translation and cross-cultural adaptation of Adolescent Asthma Self-Efficacy Questionnaire to Brazilian Portuguese and analysis of its measurement properties
Source: Clinics (Sao Paulo). 2025 Aug 26;80:100752. doi: 10.1016/j.clinsp.2025.100752 (PMC12834068; doi:10.1016/j.clinsp.2025.100752)
Supplement: Supplementary file 1 [file mmc1.docx]

**CLINICS-D-24-00594**

**Supplementary Material**

**Final version of the AASEQ**

**Esse questionário foi desenvolvido para nos ajudar a entender melhor como você enfrenta a sua asma. Por favor, escolha um número, de 0 a 100, que mais representa o seu nível de confiança/certeza para realizar cada uma das coisas descritas abaixo.**

Eu não consigo fazer Faço moderadamente Com certeza consigo fazer

Pergunta Confiança (0-100)

**Medicação**

Eu acredito que:

Eu sei como usar corretamente o meu inalador/espaçador/remédio para asma.

Eu sei quando usar meu remédio para asma.

Eu sei qual dos inaladores eu preciso usar.

Eu sei para que serve o meu inalador preventivo.

Eu sei para que serve o meu inalador de alívio.

**Controle de sintomas**

Eu acredito que:

Eu posso ser preparado para enfrentar um ataque de asma.

Eu sei como ficar calmo(a) quando estou tendo dificuldade para respirar.

Eu sei quando estou sem fôlego por causa da minha asma e não por causa do exercício físico.

Eu sei quando estou sem fôlego devido a minha asma e não porque estou em pânico.

Eu sei como controlar a minha asma quando estou com dificuldade para respirar.

Eu sei quando utilizar meu inalador para controlar uma dificuldade grave para respirar

Eu sei quando preciso ir ao hospital por causa de uma dificuldade grave para respirar

Eu sei como evitar os fatores que desencadeiam minha asma.

**Conhecimento sobre a asma**

Eu acredito que:

Eu tenho o controle da minha asma.

Eu posso praticar atividades físicas como esportes.

Eu posso ter uma vida normal.

Eu posso fazer as coisas que quero fazer.

Eu posso controlar minha asma no dia-a-dia.

**Amigos, família e escola**

Eu acredito que:

Eu posso utilizar meu inalador na frente dos meus amigos.

Eu posso usar meu inalador na frente de outras pessoas na escola.

Eu posso falar abertamente com os meus amigos sobre a minha asma.

Eu posso falar abertamente com os meus pais sobre minha asma.

Eu posso falar abertamente com o meu médico ou enfermeira sobre a minha asma.

Eu posso falar abertamente com meus professores sobre minha asma.

Eu posso pedir ajuda para os meus pais se eu estiver com dificuldade para respirar ou tendo um ataque de asma.

Eu posso pedir ajuda para os meus professores se eu estiver com dificuldade para respirar ou tendo um ataque de asma.

Eu posso pedir ajuda aos meus amigos se eu estiver com dificuldade para respirar ou tendo um ataque de asma.
